# Comparison of Oocyte Maturation Trigger Using Follicle Stimulating Hormone Plus Human Chorionic Gonadotropin versus hCG Alone in Assisted Reproduction Technology Cycles

**DOI:** 10.22074/ijfs.2019.5701

**Published:** 2019-04-27

**Authors:** Saeedeh Dashti, Abbas Aflatoonian, Nasim Tabibnejad

**Affiliations:** Research and Clinical Center for Infertility, Yazd Reproductive Sciences Institute, Shahid Sadoughi University of Medical Sciences, Yazd, Iran

**Keywords:** Follicle Stimulating Hormone Co-Trigger, Human Chorionic Gonadotropin Trigger, Pregnancy Rate

## Abstract

**Background:**

The goal of this study was to investigate oocyte maturation, fertilization and pregnancy rates among infertile women, by concomitant follicle stimulating hormone (FSH) administration at the time of human chorionic gonadotropin (hCG) trigger, compared to hCG trigger alone.

**Materials and Methods:**

In this prospective randomized controlled trial, 109 infertile women between the ages of 20 and 40 years, received gonadotropin-releasing hormone (GnRH) antagonist and fresh embryo transfer. Following the procedure, the subjects were randomly divided into two groups on the oocyte-triggering day. In the experimental group, final oocyte maturation was achieved by 5000 IU hCG plus 450 IU FSH. In the control group, however, oocyte triggering was performed by 5000 IU hCG, only. The primary outcome was clinical pregnancy and the secondary out- comes included oocyte recovery rate, oocyte maturity rate, fertilization proportion rate, fertilization rate, implantation rate and chemical pregnancy rate.

**Results:**

Fifty-four women were appointed to the group with the FSH bolus injection at the time of hCG trigger and 55 women were assigned to the hCG alone group. Women in the FSH group had a significantly higher metaphase II (MII) oocyte (7.17 ± 3.50 vs. 5.87 ± 3.19), 2 pronuclear embryos (2PNs) (5.44 ± 3.20 vs. 3.74 ± 2.30) and total em- bryos (4.57 ± 2.82 vs. 3.29 ± 2.13) compared to hCG alone group, respectively. Furthermore, fertilization rate (0.75 ± 0.19 vs. 0.68 ± 0.25), implantation rate (14.2 vs. 8.5%) as well as clinical (27.9 vs. 15.9%) and chemical (32.6 vs. 20.5%) pregnancy rates were higher in the FSH group, but no statistically significant difference was found (P>0.05).

**Conclusion:**

Combination of FSH and hCG for oocyte triggering improves oocyte maturity and fertilization propor- tion rates without increasing the chance of implantation, chemical and clinical pregnancy rates (Registration number: IRCT2017082724512N5).

## Introduction

The success rates of assisted reproduction technology (ART) have extremely improved in the recent years. This improvement is due to the new developments in laboratory techniques along with the enhancement of ovarian stimulation protocols. Several studies have investigated the efficacy of different ovarian stimulation protocols. However, the development of final oocyte maturation has not been fully evaluated (1). FSH in final oocyte triggering. It has been shown that oocyte triggering using 1500 IU hCG plus 450 IU FSH may reduce OHSS compared to the routine trigger methods such as (5000 IU hCG alone), without any positive effects on the outcomes of IVF and pregnancy (11).

The goal of this study was to compare oocyte maturation as well as fertilization and pregnancy rates among women receiving high-dose concomitant FSH administration at the time of hCG trigger to those with hCG trigger alone. We also aimed to compare OHSS development between these two groups.

## Materials and Methods

This prospective randomized controlled trial was performed at Yazd Reproductive Sciences Institute between August and November of 2017. The study was approved by the Ethics Committee of Yazd Reproductive Sciences Institute, Shahid Sadoughi University of Medical Sciences, Yazd, Iran (IR.SSU.RSI.Rec.1396.7). All couples signed a written informed consent for participation. The study was registered in Iranian Registry of Clinical Trials (IRCT2017082724512N5) and was indicated according to the CONSORT statement.

### Subjects

For this study, 109 infertile women at the ages of 20 to 40 years underwent GnRH antagonist protocol for controlled ovarian hyperstimulation and fresh embryo transfer in the same cycle. Exclusion criteria were severe male factor infertility, cycle cancellation or changing to intrauterine insemination in the subjects, and a cycle containing preimplantation genetic diagnosis (PGD) and estradiol level of more than 2500 pg/mL on the day of hCG injection. Women who did not undergo embryo transfer due to the freeze-all policy, donor, or surrogate cycle, were also excluded.

### Stimulation protocol and randomization

All patients who participated in this trial were stimulated using gonadotropins Cinnal-f (CinnaGen, Iran), which started on day 2 of the menstrual cycle. The initial dose of gonadotropin was individualized for each patient based on the age, antral follicle count (AFC), and anti-mulerian hormone (AMH) level. Gonadotropin dose adjustment was done based on ovarian response by follicular diameter measurement with transvaginal ultrasound, which was done every 2 to 3 days from the 7^th^ day of stimulation. The GnRH antagonist (Cetrorelix, Merck Serono Laboratories, Aubonne, Switzerland) was administered when the mean diameter of dominant follicles reached 13-14 mm. In all patients, oocyte triggering was performed when at least three follicles with a diameter of 18 mm or greater were found in the ultrasound examination. The participants were randomly divided into two groups on the day of trigger. Randomization was performed using computer-created random numbers in covered, unlabeled envelope each holding a single number. The patients, nurses, and physicians were not blinded to the allocated treatment groups. In the first group, final oocyte maturation was done by 5000 IU hCG (Pregnyl, Organon, Netherlands) plus 450 IU FSH (Cinnal-f Cinnagen, Iran). In the control group, oocyte triggering was performed by 5000 IU hCG alone. The dose of 450 IU FSH was chosen as it seems to be the maximum practical dose with the goal of making a FSH surge with a natural cycle (1:4-5 FSH:LH ratio) (1).

Transvaginal oocyte retrieval was done 36 hours after triggering for all subjects. Routine IVF/intracytoplasmic sperm injection (ICSI) was performed according to standard protocols (72.7% ICSI and 27.3% IVF). Oocyte maturity was assessed after cumulus cell denudation, and fertilization was evaluated 18 hours after insemination or sperm injection. The best embryos with at least 7 blastomeres (7-9 blastomeres) and a maximum of 20% cytoplasmic fragmentation were considered as grade A. Grade B embryos had 7-9 cells with over 20% fragmentation. Grade C embryos had 4-6 cells with a maximum of 20% fragmentation.

Two or three good quality embryos were transferred 48-72 hours after oocyte retrieval, using an embryo transfer Labotect catheter (Labor-TechnikGöttingen GmbH, Gottingen, Germany) or a Cook (Sydney, Australia) catheter.

### Outcome parameters

The primary outcome was clinical pregnancy, defined as the observation of fetal heart activity by transvaginal ultrasound 2-3 weeks after positive β-hCG test. Secondary outcomes included oocyte recovery rate, oocyte maturity rate, fertilization proportion rate, fertilization rate, implantation rate and chemical pregnancy, which were defined as follows: oocyte recovery rate was the number of retrieved oocytes divided by the number of follicles >10 mm in size counted on the day of trigger; oocyte maturity rate was the number of metaphase II (MII) oocytes divided by the number of oocytes retrieved; fertilization proportion was the number of 2 pronuclear (2PNs) divided by the number of oocytes retrieved; fertilization rate was the number of 2PNs divided by the total number of MII oocytes; implantation rate was the number of intrauterine gestational sacs observed by transvaginal ultrasonography divided by the total number of transferred embryos; and chemical pregnancy rate was positive β-hCG test 14 days after embryo transfer. OHSS development was also considered as a secondary outcome, so women with signs of OHSS were separated into three categories according to the signs and symptoms. Mild OHSS was considered as ovarian enlargement, lower abdominal discomfort, mild nausea, vomiting, and abdominal distention. Getting worse of symptoms, ascites, and ovarian enlargement up to 12 cm were characterized as moderate OHSS. Lastly, severe OHSS was defined by severe pain, rapid weight gain, tense ascites, hemodynamic instability, difficulty of respiration, progressive oliguria, and laboratory abnormalities (12).

### Statistical analysis

We assumed that at least a total of 100 cases are needed (50 in each group) to achieve a 15% difference in the clinical pregnancy rate as our primary outcome between the two groups. A power of 80% and P<0.05 level of significance were set for this study. The Statistical Package for the Social Science version 20 for Windows (SPSS Inc, Chicago. IL, USA) was applied for data analysis. Differences between continuous variables without normal distribution were measured by Mann-Whitney U test. The Chi-square test was used to compare categorical variables.

## Results

**Fig 1:**
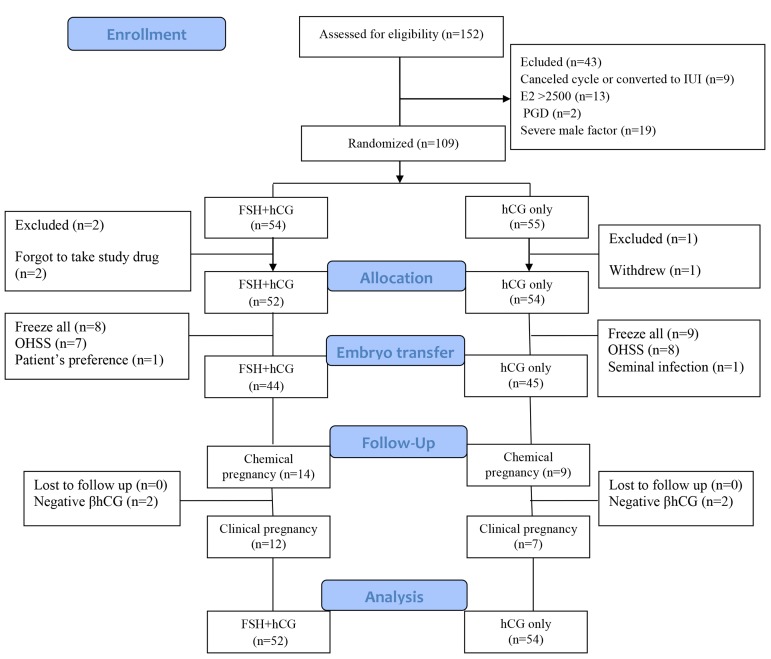
Flowchart of participants’ allocation, treatment, follow-up, and analysis. IUI; Intrauterine insemination, E2; Estrogen, PGD; Preimplantation genetic diagnosis, OHSS; Ovarian hyperstimulation syndrom, FSH; Follicle stimulating hormone, and hCG; Human chorionic gonadotropin.

Initially one hundred and fifty-two infertile women enrolled in the study. Of those, 109 participants met the inclusion criteria on the day of oocyte trigger and were randomized into two groups. Fifty-four women were appointed to the group with the FSH bolus injection at the time of hCG administration and the remaining 55 women were assigned to the hCG alone administration group. A total of 43 women who were excluded before randomization were as follows: 9 cycles were canceled or converted to intrauterine insemination, 13 women had estradiol levels more than 2500 pg/mL at the time of triggering, 2 women were planned to have PGD, 19 couples were diagnosed with severe male factor infertility, 2 women in the FSH group forgot to take the ordered medicine, and 1 patient in the control group withdrew from the treatment prior to oocyte retrieval due to personal reasons. All other participants were available throughout the study for follow-up in both groups (Fig .1). Demographics characteristics of both groups are listed in Table 1. The demographic features did not differ significantly between the two groups.

**Table 1:** Baseline characteristics of "FSH+hCG" group versus "hCG only" group


Variables		FSH+hCG n=52	hCG only n=54	P value

Age (Y)		29.84 ± 4.24	30.83 ± 4.66	0.258^*^
Duration of infertility (Y)		6.18 ± 3.69	6.05 ± 4.14	0.611^*^
Type of infertility	
	Primary	42 (80.8)	36 (66.7)	0.125^**^
	Secondary	10 (19.2)	18 (33.3)
AMH (ng/ml)		3.65 ± 2.56	4.00 ± 2.06	0.158^*^
Endometrial thickness (mm)		9.55 ± 1.72	11.30 ± 13.64	0.952^*^
Cause of infertility				0.706^**^
	Male factor	20 (38.5)	15 (27.8)
	PCOS	4 (7.7)	7 (13)
	TF	3 (5.8)	3 (5.6)
	MIX	15 (28.8)	15 (27.8)
	Unexplained	10 (19.2)	14 (25.9)


Data are presented as mean ± SD and number (%). "FSH+hCG" group versus "hCG only" group using *; Mann-Whitney U test, **; Chi-squared test, FSH; Follicle stimulating hormone, hCG; Human chorionic gonadotropin, AMH; Anti mullerian hormone, PCOS; Polycystic ovary syndrome, and TF; Tubal infertility.

Furthermore, total gonadotropin dose, serum estradiol on the day of trigger, number of total follicles on the day of trigger and number of retrieved oocytes were similar in both groups. Moreover, number and quality of the transferred embryos were comparable between the two groups. Nevertheless, women in the FSH group had a significantly higher MII oocyte, 2PNs and total embryos compared to the hCG alone group (Table 2).

**Table 2:** ART cycle characteristics of "FSH+hCG" group versus "hCG only" groups


Variables		FSH+hCGn=52	hCG onlyn=54	P value

Total gonadotropin dose (IU)		1537 ± 422	1567 ± 500	0.831^*^
Number of days of stimulation		10.08 ± 2.04	9.7 ± 1.69	0.641^*^
Serum estradiol on the day of trigger (pg/ml)		1246 ± 462	1244 ± 460	0.982^*^
Number of total follicles on the day of trigger		9.71 ± 3.21	10.16 ± 3.73	0.503^*^
Number of oocyte retrieved		8.32 ± 3.88	7.62 ± 3.93	0.322^*^
Number of MII oocytes		7.17 ± 3.50	5.87 ± 3.19	0.049^*^
Number of 2PNs		5.44±3.20	3.74 ± 2.30	0.002^*^
Number of total embryos		4.57 ± 2.82	3.29 ± 2.13	0.012^*^
Number of embryos transferred		n=441.90 ± 0.29	n=451.84 ± 0.52	0.400^*^
Quality of embryo transferred		n=44	n=45	0.231^**^
A	13 (29.5)	13 (28.8)	
B	26 (59.1)	27 (60)	
C	5 (11.4)	5 (11.1)	


Data are presented as mean ± SD and number (%). "FSH+hCG" group versus "hCG only" group using *; Mann-Whitney U test, **; Chi-squared test, ART; Assisted reproductive technology, FSH; Follicle stimulating hormone, hCG; Human chorionic gonadotropin, MII; Metaphase II, 2PN; 2 pronuclear, quality of embryos A-C as described in materials and methods.

Eight women in the FSH group and 9 women in the control group decided to use the freeze-all porcedure for future embryo transfer. Therefore, fresh embryo transfer was performed in 44 and 45 cycles in the FSH and control groups, respectively. For comparing the two treatment groups (FSH plus hCG vs. hCG only), we analyzed the reproductive outcomes such as fertilization rate (0.75 ± 0.19 vs. 0.68 ± 0.25), implantation rate (14.2 vs. 8.5%), as well as clinical (27.9 vs. 15.9%) and chemical (32.6 vs. 20.5%) pregnancy rates, which were all higher in the FSH group, but not at a statistically significant level (P>0.05). Also, we found that oocyte recovery rate showed no statistically significant difference between the two groups, however, there is a general trend towards greater oocyte recovery rate in the FSH group. In addition, women who received FSH showed significantly higher oocyte maturity rate and fertilization proportion than the other group (Table 3). Proportion of mild and moderate OHSS development was similar between the two groups (P>0.05). There was no case of severe OHSS in either group (Table 4).

**Table 3:** ART outcome of "FSH+hCG" group versus "hCG only" groups


Cariables	FSH+hCG n=44	hCG only n=45	P value

Oocyte recovery rate	0.84 ± 0.19	0.75 ± 0.26	0.066^*^
Oocyte maturity rate	0.87 ± 0.16	0.77 ± 0.19	0.004^*^
Fertilization proportion	0.65 ± 0.20	0.51 ± 0.22	0.001^*^
Fertilization rate	0.75 ± 0.19	0.68 ± 0.25	0.124^*^
Implantation rate	12/84 (14.2)	7/82 (8.5)	0.244^**^
Chemical pregnancy rate	14 (32.6)	9 (20.5)	0.231^**^
Clinical pregnancy rate	12 (27.9)	7 (15.9)	0.203^**^


Data are presented as mean ± SD and number (%). "FSH+hCG" group versus "hCG only" group using *; Mann-Whitney U test, **; Chi-squared test, ART; Assisted reproductive technology, FSH; Follicle stimulating hormone, and hCG; Human chorionic gonadotropin.

**Table 4:** OHSS occurrence in "FSH+hCG" group versus "hCG only" groups


OHSS occurrence	FSH+hCG n=52	hCG only n=54	P value

No OHSS	45 (86.5)	46 (85.2)	0.968
Mild	5 (9.6)	6 (11.1)	
Moderate	2 (3.8)	2 (3.7)	
Severe	--	--	


Data are presented as number (%). "FSH+hCG" group versus "hCG only" group using Chi-squared test. FSH; Follicle stimulating hormone, hCG; Human chorionic gonadotropin, and OHSS; Ovarian hyper stimulation syndrome.

## Discussion

Our results showed that co-administration of a bolus dose of FSH and hCG for oocyte triggering improves the number of MII oocytes, 2 PNs, embryos, as well as oocyte maturity rate and fertilization proportion, compared to hCG injection only.

It is well-documented that mid-cycle LH surge is necessary for oocyte maturation, but the possible alternative is poorly investigated. Animal studies have indicated that FSH is capable of inducing ovulation. In hypophysectomized rats ovulation was induced using LH-free recombinant FSH, and as a result a 100% dose-dependent ovulation rate was obtained (13). Similarly, another study found comparable ovulation rates in both FSH-stimulated and hCG-induced mice (14). Moreover, Zelinski-Wooten et al. (15) showed that a single bolus of 2500 IU recombinant FSH was equivalent to 1000 IU hCG for induction of mid-cycle FSH surge, meiosis resumption and fertilization in rhesus monkeys. The first human report of FSH injection at the time of oocyte trigger was a case study, which administered 1050 IU FSH instead of standard 10000 IU hCG before oocyte retrieval. This case report indicated that administration of recombinant human FSH results in production of good quality oocyte with the maturity rate of 90% and consequent high-graded embryos. Although no pregnancy was achieved after transfer of fresh or frozen-thawed embryos in this study (2). 

Similar to our findings, in another study the authors triggered oocytes by adding either 450 IU FSH injection or normal saline as placebo at the time of hCG administration. The results showed a significant rise of oocyte recovery rate and fertilization proportion in FSH group compared to the placebo-treated group. Furthermore, we found higher fertilization and implantation rates along with chemical and clinical pregnancies, but the difference did not reach significance. In the same way, Lamb and colleagues, reported insignificant increase in implantation rate, clinical and ongoing pregnancy rates. Although they did not assess oocyte maturity rate, but their IVF fertilization rate was reported significantly higher in the FSH group (1).

Mid-cycle FSH surge promotes oocyte cumulus expansion and oocyte nuclear maturation through constitution of signaling pathways and bilateral communications between oocytes and cumulus cells by opening the gap junctions (16). On the other hand, FSH induces plasminogen activator gene expression (13) as well as elevation of plasminogen activator (17, 18). In primates, FSH facilitates detachment of oocytes from follicular wall and provokes follicular rupture (19). In rodents, this possibility is confirmed by an increase in oocyte recovery following a bolus injection of recombinant FSH (20), which is similar to that in humans (1, 21). In agreement with aforementioned studies, we found higher oocyte recovery rate in the FSH group compared to the control women.

Several studies have shown indirect beneficial effects of FSH on the occurrence of OHSS. For instance, using GnRHa for final oocyte maturation minimizes the chance of OHSS, with comparable results than conventional oocyte triggering (6, 22-24). Nonetheless, an important disadvantage of GnRHa triggering is failure to induce LH surge in downregulated stimulation cycles in patients with hypothalamic dysfunction. Oocyte triggering by low dose hCG plus FSH, however, could be beneficial in all types of stimulation protocols. We also compared OHSS development in the two study groups and found similar rates between FSH plus hCG and hCG alone groups. We know of only one study, in which the authors assessed the effects of FSH for oocyte triggering in high responder women. They applied 1500 IU hCG plus 450 IU FSH for OHSS prevention and found that OHSS associated symptoms were significantly lower among women receiving FSH and hCG compared to those treated with hCG alone. Moreover, the authors claimed that this strategy induced meiosis resumption and cytoplasmic maturation, which resulted in high quality oocytes with competence to generate normal embryos leading to live births (11).

## Conclusion

Our results showed that adding 450 IU FSH to 5000 IU hCG for oocyte triggering improves oocyte maturity and fertilization proportion rates. However, it does not increase the chance of successful implantation or chemical and clinical pregnancy rates. Prominently, further studies are required to optimize this novel triggering strategy with regards to concentration, sample size, etc. to provide significantly higher pregnancy percentages along with reduced OHSS rate.
